# The Influence of Glucagon-like Peptide-1 Receptor Agonists and Other Incretin Hormone Agonists on Body Composition

**DOI:** 10.3390/ijms262412130

**Published:** 2025-12-17

**Authors:** Lampros Chrysavgis, Niki Gerasimoula Mourelatou, Maria-Evangelia Koloutsou, Sophia Rozani, Evangelos Cholongitas

**Affiliations:** 1 First Department of Internal Medicine, Medical School, National and Kapodistrian University of Athens, General Hospital Laiko, 115 27 Athens, Greece; lchrisaugis@gmail.com (L.C.); nikimourelatou@yahoo.gr (N.G.M.); sofrozan@gmail.com (S.R.); 2 First Department of Propaedeutic Internal Medicine, Medical School, National and Kapodistrian University of Athens, General Hospital Laiko, 115 27 Athens, Greece; mkoloutsou@yahoo.com

**Keywords:** glucagon-like peptide-1 (GLP-1), incretin hormones, body weight, sarcopenia, sarcopenic obesity, myosteatosis, lean mass, type 2 diabetes mellitus, metabolic dysfunction-associated steatotic liver disease (MASLD)

## Abstract

Glucagon-like peptide-1 receptor agonists (GLP-1 RAs) and newer incretin-based co-agonists have transformed obesity and type 2 diabetes (T2D) management, achieving unprecedented weight loss and cardiometabolic benefits. However, their effects on body composition, particularly lean and skeletal muscle mass, remain incompletely defined. In this current review, we examined the influence of GLP-1 RAs and incretin hormone agonists on lean tissue, integrating physiological, clinical, and mechanistic perspectives. We first outlined the physiology of incretin hormones, with emphasis on their metabolic roles and potential relevance to muscle health. We then discussed sarcopenia and sarcopenic obesity as conditions of rising clinical concern, given their overlap with obesity and metabolic disease. Evidence from preclinical studies and randomized clinical trials indicates that while GLP-1-based therapies predominantly reduce adipose tissue, including visceral and ectopic depots, but they also produce absolute reductions in lean mass, generally representing 20–30% of total weight loss. The extent to which these changes translate into impaired muscle function or increased vulnerability to frailty remains unclear. Preservation of lean and skeletal muscle mass is a critical yet underexplored aspect of incretin-based weight loss. Current studies are constrained by methodological heterogeneity, small sample sizes, and limited assessment of functional outcomes. Data on dual and triple agonists are emerging but remain limited. Future research should integrate standardized body-composition measures, mechanistic exploration, and adjunctive interventions such as resistance training or protein optimization.

## 1. Introduction

Over the past decade, glucagon-like peptide-1 receptor agonists (GLP-1 RAs) have emerged as a transformative class of pharmacotherapies for obesity and type 2 diabetes mellitus (T2D) [1,2]. Obesity represents a major global health challenge, affecting more than 650 million adults worldwide and contributing substantially to morbidity, mortality, and healthcare costs [3]. Beyond excess adiposity, obesity is characterized by complex alterations in body composition, including the accumulation of visceral and ectopic fat, infiltration of adipose tissue into muscle and liver, and progressive impairment of skeletal muscle quality [4,5]. Large randomized controlled trials (RCTs) have demonstrated that GLP-1 RAs such as liraglutide [6] and semaglutide [7] produce clinically meaningful, durable weight loss and reduction in adipose tissue, especially visceral and ectopic depots alongside improvements in glycaemic control and cardiovascular outcomes. More recently, dual and triple incretin agonists, targeting combinations of GLP-1, glucose-dependent insulinotropic polypeptide (GIP), and glucagon (GCG), have achieved even greater weight reductions in clinical trials, raising the prospect of unprecedented efficacy in medical obesity treatment [8,9,10].

However, the profound reductions in body weight achieved with incretin-based therapies have renewed attention to their effects on body composition. Weight loss achieved through lifestyle or surgical interventions is often accompanied by decreases in both fat mass and lean mass [11], with lean tissue typically accounting for 20–30% of total weight reduction [8]. Loss of skeletal muscle mass may have important implications for metabolic health, functional capacity, and long-term outcomes, particularly in older adults and those with comorbidities. Whether incretin-based therapies preserve, disproportionately reduce, or even improve aspects of lean tissue and muscle quality remains an open and clinically relevant question [12]. Moreover, interindividual variability, methodological heterogeneity in body-composition assessment, and limited data on long-term outcomes complicate interpretation. The addition of lifestyle strategies, such as resistance exercise and protein optimization, may offer opportunities to enhance muscle preservation during pharmacologically induced weight loss [13,14], but rigorous trial evidence remains scarce.

Thus, while the efficacy of incretin-based therapies in achieving weight loss is firmly established, their impact on lean muscle mass remains insufficiently explored [15], underreported, and underrepresented in current clinical guidelines. This gap is particularly important given the aging population, where interventions that preserve muscle health may critically influence long-term outcomes [16].

This review aims to synthesize current knowledge on the influence of GLP-1 RAs and incretin hormone agonists on body composition. We summarized the physiologic actions of GLP-1, GIP and GCG and their association with metabolic disorders. Furthermore, we discussed evidence from preclinical studies on the effect of GLP-1 RAs and co-agonists on body composition parameters, highlighting the pathogenetic mechanisms implicated in this process. Of importance, we emphasized the published RCTs regarding the influence of incretin hormone agonists on body weight, lean muscle mass as well as we pinpointed methodological considerations and potential limitations, thus exploring emerging insights into mechanisms. Lastly, this review considers emerging strategies aimed at mitigating lean mass loss, such as combining incretin-based therapies with structured exercise programs or employing adjunctive anabolic approaches including myostatin/ActRII inhibition, to provide a broader perspective on muscle preservation during pharmacologically induced weight loss.

## 2. Physiology of Incretin Hormones

### 2.1. Glucagon-like Peptide 1 (GLP-1)

**Figure 1:**
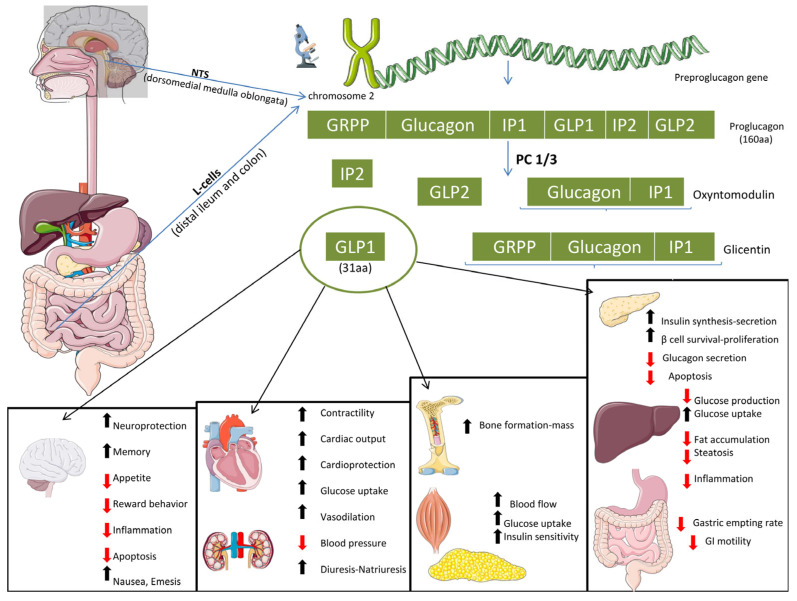
Physiology and multi-organ effects of glucagon-like peptide-1 (GLP-1). Following nutrient stimulation, GLP-1 acts via its receptor in multiple organs to enhance insulin secretion and β-cell survival, suppress glucagon release, and reduce hepatic gluconeogenesis, steatosis, and inflammation. In muscle and adipose tissue, it improves glucose uptake and insulin sensitivity, while in the cardiovascular system it promotes vasodilation and cardioprotection. GLP-1 also regulates appetite, energy balance, and neuroprotection, integrating glycaemic control with systemic metabolic and organ benefits. Abbreviations: aa, amino acids; AMPK, AMP-activated protein kinase; Akt (PKB), protein kinase B; AGEs, advanced glycation end-products; ActRII, activin receptor type II; β-cell, pancreatic beta cell; cAMP, cyclic adenosine monophosphate; CREB, cAMP response element-binding protein; DIO, diet-induced obesity; DPP-4, dipeptidyl peptidase-4; ERK, extracellular signal-regulated kinase; FFAs, free fatty acids; FGF-21, fibroblast growth factor-21; GCG, glucagon; GIP, glucose-dependent insulinotropic polypeptide; GIPR, glucose-dependent insulinotropic polypeptide receptor; GI, gastrointestinal; GLP-1, glucagon-like peptide-1; GLP-1R, glucagon-like peptide-1 receptor; GLP-2, glucagon-like peptide-2; GRPP, glicentin-related pancreatic peptide; HbA1c, glycated haemoglobin; HCC, hepatocellular carcinoma; IL, interleukin; IP-1/IP-2, intervening peptide-1/intervening peptide-2; IR, insulin resistance; L-cells, enteroendocrine L-cells; MAPK, mitogen-activated protein kinase; MASLD, metabolic dysfunction-associated steatotic liver disease; MASH, metabolic dysfunction-associated steatohepatitis; MHC, myosin heavy chain; MyoD, myoblast determination protein; NTS, nucleus tractus solitarius; PC 1/3, prohormone convertase 1/3; PI3K, phosphoinositide 3-kinase; PPAR, peroxisome proliferator-activated receptor; ROS, reactive oxygen species; SIRT1, sirtuin-1; SVR, skeletal muscle-to-visceral fat ratio; T2D, type 2 diabetes; TCA, tricarboxylic acid; TNF-α, tumour necrosis factor-alpha. (Created with BioRender.com). Arrow and color definition: ↑ indicates increase/enhancement; ↓ indicates decrease/suppression. Black arrows denote physiological or beneficial actions, while red arrows indicate pathological processes reduced or inhibited by GLP-1.

Glucagon-like Peptide 1 (GLP-1) (Figure 1) is a 30-amino-acid polypeptide derived from the cleavage of proglucagon, a 160-amino-acid precursor polypeptide encoded by the *Gcg* gene located on chromosome 2 [17,18,19]. GLP-1 secretion is triggered by the ingestion of mixed meals—comprising glucose, fatty acids, amino acids, and dietary fibre—and follows a biphasic pattern: an early phase occurring within 10–15 min, and a prolonged phase lasting 30–60 min. The early phase is primarily regulated by the autonomic nervous system, particularly the vagus nerve, with acetylcholine and gastrin-releasing peptide (GRP) acting as key neurotransmitters. The delayed phase is mediated by direct nutrient contact with L-cells and influenced by hormones like leptin [20,21,22]. GLP-1 is rapidly degraded in circulation, primarily by the enzyme dipeptidyl peptidase-IV (DPP-IV), which removes the two N-terminal amino acids, generating GLP-1 (9–36) amide or GLP-1 (9–37). These are considered as inactive forms that may act as weak competitive antagonists at the GLP-1 receptor (GLP-1R), although no antagonistic effects have been observed in vivo [23,24]. Due to this rapid degradation, only about 25% of secreted GLP-1 remains in its active form by the time it reaches the liver, where an additional 40–50% is metabolized. Ultimately, only 10–15% of active GLP-1 enters systemic circulation intact [25]. GLP-1 is a key incretin hormone that enhances insulin secretion in response to oral glucose intake, a phenomenon known as the incretin effect. This effect underscores its critical role in postprandial glucose regulation and insulin release [26,27,28]. In healthy individuals, GLP-1 contributes to approximately 70% of insulin secretion following a meal, a proportion that decreases to around 30% in individuals with T2D [29].

### 2.2. Glucose-Dependent Insulinotropic Polypeptide (GIP)

**Figure 2:**
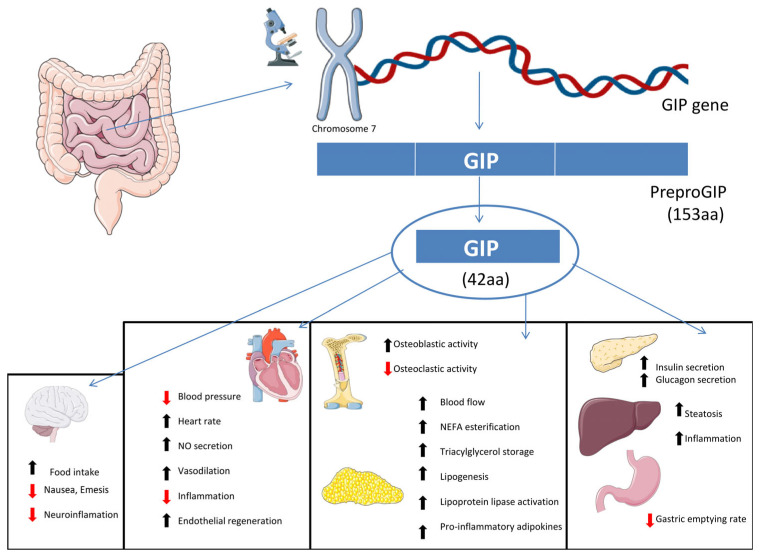
Biosynthesis and physiological actions of glucose-dependent insulinotropic polypeptide (GIP). GIP is secreted in response to nutrient ingestion, and it enhances insulin and glucagon secretion, promotes osteoblastic activity, and modulates lipid metabolism by stimulating lipogenesis, NEFA esterification, and lipoprotein lipase activation. GIP also regulates appetite and neuroinflammation, increases endothelial regeneration, and exerts anti-atherogenic and vasodilatory effects. Abbreviations: aa, amino acids; GIP, glucose-dependent insulinotropic polypeptide; NEFA, non-esterified fatty acids; NO, nitric oxide. (Created with BioRender.com). Arrow and color definition: ↑ indicates increase/enhancement; ↓ indicates decrease/suppression. Black arrows denote physiological or beneficial actions, while red arrows indicate pathological processes reduced or inhibited by GIP.

Glucose-dependent insulinotropic polypeptide (GIP) (Figure 2) was the first incretin hormone to be identified, with its physiological role being elucidated in 1973 [30]. GIP is a 42-amino-acid peptide derived from a 153-amino-acid precursor, proGIP, via post-translational cleavage mediated by prohormone convertase 1/3 (PC1/3) at specific single-arginine residues [31]. Following glucose or mixed-meal ingestion, GIP levels peak within 30 to 60 min, with the response magnitude influenced by meal composition. Beyond its classical incretin function, GIP promotes glucagon secretion from pancreatic α-cells, particularly during hypoglycaemia in healthy individuals [32]. Supraphysiological doses of long-acting GIP agonists have demonstrated neuroprotective and anti-inflammatory effects in models of neurodegenerative diseases, through the suppression of microglial and astrocyte activation [33]. GIP also promotes vascular and endothelial health as it can stimulate endothelial regeneration and exerts anti-atherosclerotic effects by activating GIP-receptor (GIPR) signalling, which inhibits foam cell formation and macrophage infiltration [34,35]. Additionally, GIP enhances nitric oxide (NO) production in endothelial cells, contributing to vasodilation, reduced inflammation, and protection against adverse arterial remodelling [36]. In lipid metabolism, GIP contributes to the expansion and healthy function of white adipose tissue by increasing tissue perfusion, enhancing non-esterified fatty acid (NEFA) esterification, and promoting triacylglycerol storage [37]. Elevated GIP levels, particularly in obesity, are associated with increased hepatic fat accumulation, elevated markers of liver injury, and increased plasma levels of fibroblast growth factor 21 (FGF-21), a marker of metabolic stress [38]. Furthermore, altered hepatic microRNA expression linked to GIP signalling suggests epigenetic involvement in liver steatosis [39,40].

### 2.3. Glucagon (GCG)

**Figure 3:**
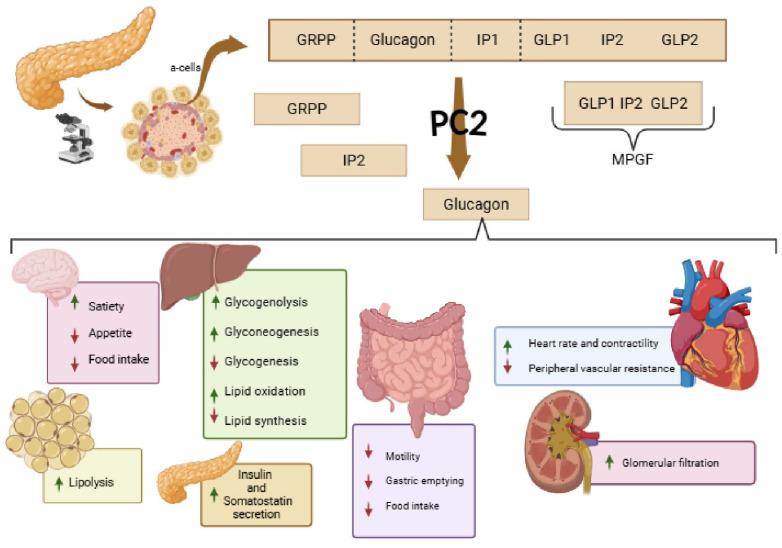
Biosynthesis and multi-organ actions of glucagon. Upon nutrient deprivation or hypoglycaemia, glucagon stimulates hepatic glycogenolysis, gluconeogenesis, lipid oxidation, and ketogenesis while inhibiting lipogenesis. It enhances insulin and somatostatin secretion, promotes lipolysis in adipose tissue, and regulates gastric motility and food intake. In the cardiovascular system, glucagon increases heart rate and contractility, and in the kidney, it modulates glomerular filtration. Abbreviations: α-cells, pancreatic alpha cells; GLP-1, glucagon-like peptide-1; GLP-2, glucagon-like peptide-2; GRPP, glicentin-related pancreatic peptide; IP1/IP2, intervening peptide-1/intervening peptide-2; MPGF, major proglucagon fragment; PC2, prohormone convertase 2. (Created with BioRender.com). Arrow and color definition: ↑ indicates increase/enhancement; ↓ indicates decrease/suppression. Green arrows denote physiological or beneficial actions, while red arrows indicate pathological processes reduced or inhibited by glucagon.

Glucagon (GCG) (Figure 3) was identified in 1923 and is a 29–amino acid peptide hormone essential for glucose homeostasis [41]. Tissue-specific processing by prohormone convertases yields glucagon in pancreatic α-cells (via PC2) and GLP-1/GLP-2 in intestinal L-cells (via PC1/3) [42]. Glucagon acts through the glucagon receptor (GCGR), a class B G protein-coupled receptor highly expressed in the liver and, to a lesser extent, in adipose tissue, kidney, heart, and the central nervous system [43]. GCGR activation increases cyclic AMP (cAMP), stimulating protein kinase A (PKA) and downstream transcriptional regulators such as cAMP response element-binding protein (CREB) [44], thereby promoting hepatic glycogenolysis, gluconeogenesis, and fatty acid oxidation [45]. Beyond glucose regulation, glucagon increases energy expenditure by stimulating thermogenesis and lipid oxidation in liver and muscle [46]. Chronic hyperglucagonemia, as seen in T2D and obesity, may promote fasting hyperglycaemia, hepatic steatosis, and muscle catabolism [47].

## 3. Main Dysregulations in Body Composition

### 3.1. Sarcopenia and Sarcopenic Obesity (SO)

SO—derived from the Greek *sarx* (flesh) and *penia* (loss)—is a progressive skeletal muscle disorder characterized by the loss of muscle mass and function, a term first introduced by Rosenberg in 1989 [48,49]. According to the European Working Group on Sarcopenia in Older People (EWGSOP), a diagnosis of sarcopenia requires concurrent findings of low muscle mass, reduced muscle strength, and poor physical performance [50]. This condition disproportionately affects the elderly and is associated with increased risk of falls, fractures, post-operative complications, and overall mortality [51,52]. A large meta-analysis estimates sarcopenia prevalence in older adults at 10–16%, a significantly higher rate than in the general population [53]. However, the disease also manifests in younger populations, often associated with physical inactivity, malnutrition, chronic diseases, and metabolic disorders such as T2D and metabolic dysfunction-associated steatotic liver disease (MASLD), the latest term for the disease formerly known as non-alcoholic fatty liver disease (NAFLD) [54,55].

Obesity, defined by the World Health Organization (WHO) as abnormal or excessive fat accumulation, is diagnosed when body mass index (BMI) is ≥30 kg/m^2^ in adults. For Asian populations, a lower threshold of ≥27.5 kg/m^2^ is recommended due to elevated obesity-related health risks at lower BMIs [56]. The coexistence of sarcopenia and obesity—referred to as SO—was first described by Baumgartner et al. in 2000 [57]. Globally, SO affects approximately 11% of older adults and up to 23% of individuals aged ≥ 75 years [58]. This condition is projected to affect as many as 200 million people globally by 2050 [59]. In 2022, the European Society for Clinical Nutrition and Metabolism (ESPEN) and the European Association for the Study of Obesity (EASO) proposed diagnostic criteria for SO [57]. The diagnostic pathway commences with the identification of individuals presenting with increased BMI or waist circumference, together with clinical or functional indicators suggestive of sarcopenia. A positive screening subsequently prompts confirmatory assessments of muscle composition and performance [57,60]. SO is a complex syndrome influenced by multiple factors, including aging, undernutrition, physical inactivity, systemic inflammation, hormonal imbalances, and insulin resistance [61]. This condition contributes to an increased risk of metabolic diseases such as T2D and MASLD, reduces quality of life, and is associated with increased morbidity and mortality [61,62].

### 3.2. Myosteatosis

Myosteatosis refers to the pathological deposition of fat within muscle tissue. This lipid infiltration may occur in several forms: between muscles (intermuscular adipose tissue), within muscle fibres (intramuscular adipose tissue), and inside muscle cells as lipid droplets (intramyocellular lipids) [63]. Diagnosis is predominantly based on non-invasive imaging modalities, particularly computed tomography (CT), given that skeletal muscle biopsy is rarely performed [64,65]. Myosteatosis is often linked to impaired mitochondrial lipid oxidation, muscular dystrophies, and various metabolic disorders [66]. Importantly, it is not merely an ectopic fat depot; it negatively impacts muscle mobility, contractility, and performance. Moreover, myosteatosis correlates with insulin resistance and other metabolic abnormalities, positioning it as a potential early biomarker for metabolic disease risk [65].

## 4. Muscle Dysregulations and Their Impact on Metabolic Syndrome and MASLD (Table 1)

Skeletal muscle disorders are closely interrelated with key metabolic diseases and comorbidities such as T2D. These conditions share overlapping pathophysiological mechanisms, including insulin resistance, chronic inflammation, oxidative stress, and lipid dysregulation [67]. The prevalence of sarcopenia is significantly higher in T2D patients compared to normoglycemic individuals, reaching up to 29.3% [68]. Likewise, SO is prevalent in about 27% of individuals with T2D [69,70]. Of importance, the association between T2D and muscle disorders is bidirectional [71,72,73]. Sarcopenia can contribute to the onset of T2D by reducing skeletal muscle mass, a major site for insulin-mediated glucose disposal, while sarcopenia is also associated with physical inactivity and frailty, both risk factors for T2D [73,74,75]. Of note, SO may also have a synergistic impact on metabolic deterioration. In T2D, SO has been associated with impaired renal function, macroalbuminuria, cognitive decline, and cardiovascular disease [69,76]. Myosteatosis is also independently linked to insulin resistance and hyperinsulinemia, even after adjusting for total fat and regional adiposity, underscoring its metabolic impact [77]. Recognizing myosteatosis as an early marker of T2D may contribute to the development of novel predictive models for metabolic risk [65,78,79]. These conditions are interrelated and mutually reinforcing excess adiposity impairs mobility and induces chronic inflammation, insulin resistance, and oxidative stress, all of them contributing to accelerated muscle degeneration [57]. Conversely, sarcopenia promotes obesity by reducing physical activity and energy expenditure [57]. Patients with SO exhibit higher risks of cardiometabolic diseases and mortality [59,80], with outcomes generally worse than when either condition occurs on its own [59]. In obese individuals undergoing bariatric surgery, pre-existing myosteatosis may influence the procedure’s effectiveness, affecting weight loss outcomes and metabolic improvements [81].

MASLD is deeply associated with metabolic dysfunctions, including insulin resistance, obesity, and T2D [82]. The prevalence of myosteatosis in MASLD patients is estimated at approximately 27.6%, significantly higher than in healthy adults [83,84]. Sarcopenia and myosteatosis are more prevalent in patients with advanced liver disease [84]. However, myosteatosis often develops independently and prior to sarcopenia, suggesting its potential utility as an earlier and more sensitive biomarker [63,85]. Importantly, severe myosteatosis—not sarcopenia—has been specifically linked to early stages of metabolic dysfunction–associated steatohepatitis (MASH), suggesting its utility as a novel biomarker for disease progression and fibrosis risk [86]. Both sarcopenia and myosteatosis are associated with increased MASLD severity and mortality [63,84,85]. Central obesity combined with low muscle mass are associated with a higher prevalence of MASLD [87], while they were considered as significant independent predictors of cardiovascular disease (CVD) and may serve as useful tools for 10-year CVD risk stratification in MASLD patients [87]. These muscle abnormalities are strongly associated with increased frailty, complications, and mortality [88]. Identifying them early, particularly through advanced imaging and validated biomarkers, may facilitate better risk stratification, disease management, and therapeutic intervention. Further research is essential to unravel the complex interplay between muscle health and metabolic dysfunction. A deeper understanding of shared mechanisms could pave the way for novel predictive tools and improve clinical outcomes through integrated metabolic-muscular care strategies [64].

## 5. GLP-1, GIP and GCG Receptor Agonists and Their Impact on Body Composition and Lean Mass

**Figure 4:**
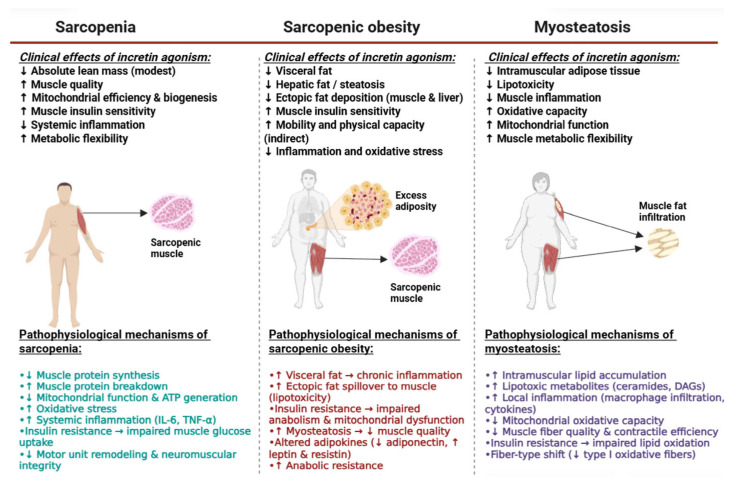
Pathophysiological mechanisms and influence of incretin-based therapies on sarcopenia, sarcopenic obesity, and myosteatosis. This figure summarizes the clinical effects of GLP-1 receptor agonists and dual/triple incretin agonists on three interconnected muscle conditions. In sarcopenia, incretin agonism modestly reduces lean mass but improves muscle quality, mitochondrial efficiency, insulin sensitivity, inflammation, and metabolic flexibility. In sarcopenic obesity, these agents reduce visceral and ectopic fat, improve metabolic inflammation, and enhance muscle insulin sensitivity. In myosteatosis, incretin therapies lower intramuscular fat and lipotoxicity while improving mitochondrial function and oxidative capacity. The bottom panels outline the key pathophysiological mechanisms underlying each condition. Abbreviations: ATP, adenosine triphosphate; DAGs, diacylglycerols; GLP-1, glucagon-like peptide-1; GIP, glucose-dependent insulinotropic polypeptide; IL-6, interleukin-6; TNF-α, tumour necrosis factor-alpha. ↑ indicates increase/enhancement; ↓ indicates decrease/suppression. (Created with BioRender.com).

Evidence based on experimental preclinical models (Figure 4).

Preclinical studies indicate that GLP-1RAs and co-agonists exert complex and sometimes divergent effects on skeletal muscle composition and function, reflecting the involvement of multiple and distinct pathophysiological mechanisms (Table 2).

In murine models, semaglutide elicited an increase in the cross-sectional area of tibialis anterior and gastrocnemius fibres, accompanied by an overall decline in total muscle weight. These findings suggest a disproportionate preservation of myofibre integrity relative to total lean mass, indicative of adaptive hypertrophic remodelling under GLP-1 receptor activation [89,90]. Co-administration with the activin receptor type II (ActRII) inhibitor bimagrumab preserved fibre morphology, implying that ActRII blockade may synergize with GLP-1RAs to mitigate lean mass loss [91]. In diet-induced obesity (DIO) models, both liraglutide and semaglutide reduced relative lower limb muscle weight compared with chow-fed controls but attenuated high-fat-diet–induced atrophy, improving fibre organization, contractility, and resistance to lipotoxic infiltration [92]. Beyond these structural effects, GLP-1RAs enhanced muscle microvascular perfusion and capillary recruitment by improving nutrient and insulin delivery to myocytes, which may support muscle energy metabolism and limit proteolytic stress [93]. Semaglutide-treated rats showed marked reductions in adiposity while maintaining lean tissue, whereas vehicle-treated controls accumulated fat and lost muscle mass. These findings support a dual mechanism by which GLP-1RAs preferentially mobilize adipose stores while partially preserving skeletal muscle integrity [92,94].

At the metabolic level, GLP-1RAs modulate substrate utilization within skeletal muscle. Liraglutide increased acetyl-CoA availability and enhanced de novo fatty acid synthesis in DIO models while suppressing glycolysis and amino acid catabolism, thereby reflecting a shift toward lipid-based energy metabolism and altered mitochondrial substrate preference [95]. GLP-1RA treatment also promotes mitochondrial biogenesis and remodelling, increasing mitochondrial density, citrate-synthase activity, and oxidative phosphorylation efficiency [96]. This shift toward oxidative metabolism is often accompanied by fibre-type remodelling, with enrichment of type I (oxidative) fibres and improved endurance capacity in GLP-1 overexpression models [97]. Transcriptomic profiling corroborates this metabolic reprogramming: RNA sequencing of gastrocnemius muscle from GLP-1 overexpression models revealed differential expression of over 700 genes enriched in AMPK phosphorylation, phosphoinositide 3-kinase (PI3K–Akt), cAMP-mediated signalling, calcium homeostasis, tricarboxylic acid cycle (TCA) cycle, phospholipase D metabolism, and chemokine signalling pathways [95,97]. Collectively, these changes indicate that GLP-1RAs activate integrated anabolic and metabolic signalling networks that contribute to muscle functional preservation [97].

At the cellular level, GLP-1RAs directly stimulate myogenesis and suppress proteolytic and inflammatory cascades. In C2C12 myoblasts, liraglutide promoted differentiation via a cAMP–PKA–dependent cascade involving PI3K/Akt, p38 MAPK, and ERK pathways. This was accompanied by upregulation of the myogenic transcription factors MyoD, myogenin, and CREB, key regulators of hypertrophy, repair, and regeneration [98]. In parallel, GLP-1RAs downregulated atrogenes such as Atrogin-1 and MuRF-1, thereby limiting ubiquitin–proteasome-mediated protein degradation [99]. In denervation-induced atrophy models, liraglutide preserved myotube morphology and reduced proteolysis, though this protection was attenuated by concurrent glucocorticoid exposure [98]. Furthermore, GLP-1RAs attenuated intramuscular inflammation by reducing Tumour necrosis factor alpha (TNF-α), interleukin (IL)-6, and IL-1β expression, and enhanced antioxidant defences via SIRT1 activation and increased superoxide dismutase activity [99]. Collectively, these data suggest that GLP-1RAs support skeletal muscle health through systemic fat reduction, direct pro-myogenic signalling, and metabolic reprogramming that enhances mitochondrial efficiency and contractile capacity [97,98,99].

### 5.1. ActRII Blockade and the Myostatin Pathway

The activin receptor type II (ActRII) pathway is a key negative regulator of skeletal muscle growth, mediating the inhibitory actions of myostatin and activin A on protein synthesis. Pharmacologic ActRII blockade promotes hypertrophy by suppressing proteolytic activity and activating anabolic pathways [91]. Co-administration of bimagrumab with semaglutide restored muscle fibre size and architecture to levels comparable with untreated controls, effectively counteracting GLP-1RA–associated lean mass reductions [91]. In murine models, ActRIIB inhibition augmented hypertrophy, particularly when combined with dietary protein, through stimulation of protein synthesis and suppression of ubiquitin–proteasome–mediated degradation [91,100]. Mechanistically, ActRII inhibition engages Akt-independent anabolic signalling, enabling muscle accretion even under conditions of systemic weight loss [97,100,101]. Beyond structural preservation, ActRII blockade improves glucose tolerance and lipid handling, underscoring its potential dual benefit in obesity and metabolic syndrome [8].

### 5.2. Dual and Triple Incretin Agonists (GLP-1, GIP, Glucagon)

Next-generation incretin-based therapies—including dual and triple agonists targeting GLP-1, GIP, and/or GCG receptors—can achieve greater overall weight reduction than GLP-1RAs alone, primarily through adipose loss while sparing lean mass [102]. GLP-1/GIP co-agonists exert synergistic effects on satiety and energy expenditure, partly through enhanced mitochondrial fatty-acid oxidation and improved muscle insulin sensitivity, although minor reductions in lean tissue have been reported in some transcriptomic studies [97]. Tri-agonists activating GLP-1/GIP/GCG pathways further enhance insulin sensitivity and lipid oxidation compared with GLP-1 monotherapy while limiting lean tissue depletion relative to caloric restriction alone [103,104]. These effects likely reflect coordinated activation of anorexigenic circuits, enhanced lipid catabolism, and partial preservation of muscle protein synthesis [104].

## 6. GLP-1 RAs, GIP and GCG Receptor Agonists and Their Efficacy on Body Composition, Skeletal and Lean Mass: Evidence from Randomized Clinical Trials (RCTs)

The therapeutic landscape of obesity and T2D has been profoundly transformed by the emergence of incretin-based pharmacotherapies, particularly GLP-1 RAs and, more recently, dual and triple agonists targeting GLP-1, GIP, and GCG receptors. While the metabolic benefits of these agents, especially their potent effects on glycaemic control and body weight are well established, their impact on body composition, and specifically on lean body mass, remains a topic of considerable scientific and clinical interest. A key concern in pharmacological weight reduction is the potential loss of fat-free mass, which includes skeletal muscle, a critical determinant of insulin sensitivity, physical function, and metabolic health, as we previously highlighted in experimental models. In individuals with obesity or sarcopenia-prone phenotypes (e.g., older adults, patients with T2D), disproportionate lean mass loss during treatment may lead to impaired glucose utilization, frailty, and adverse cardiovascular outcomes.

We encompassed all the published RCTs (Table 3) that have assessed the impact of GLP-1 RAs as well as double/triple agonists on body composition. The included RCTs utilized a range of body composition assessment methods—most commonly dual-energy X-ray absorptiometry (DXA), followed by bioelectrical impedance analysis (BIA) and, in fewer cases, magnetic resonance imaging (MRI). Across these 22 RCTs, interventions ranged from short-term (8 weeks) to long-term (up to 72 weeks), as did study populations, including individuals with obesity, T2D, prediabetes and NAFLD/MASLD, with sample sizes from 15 to 371 participants. The majority focused on liraglutide (in doses ranging from 0.6 to 3.0 mg/day), with others evaluating semaglutide, exenatide, and tirzepatide.

More specifically, in Astrup et al. [105] study, liraglutide 3.0 mg led to a total weight loss of approximately 8.0 kg over 20 weeks, with 1.5 kg attributable to lean mass loss, representing ~18% of total weight lost. Consistently, in the studies of Silver et al. [112] and Kadouh et al. [118] administration of liraglutide 1.8–3.0 mg over short durations (14–16 weeks) was examined. Both studies reported modest lean mass reductions (~1.1–1.2 kg), with relative preservation of lean mass when combined with behavioural support. In contrast, Neeland et al. [6] observed a greater lean mass reduction (2.3 kg) over 49 weeks, also using liraglutide 3.0 mg, however accompanied by a larger fat mass loss (~6.2 kg). Interestingly, two other studies [119,120] reported similar lean mass losses (~1.8–2.0 kg) using liraglutide 3.0 mg over 16 to 48 weeks. These data suggest that longer intervention durations may lead to more significant absolute lean mass reductions, possibly attributed to greater body weight reduction, although the proportion of lean-to-fat loss remains relatively stable.

Studies using semaglutide, by Blundell et al. [121] and McCrimmon et al. [122] reported lean mass losses of 1.3–1.7 kg over 12 to 52 weeks. In both trials, participants experienced >10% total weight loss, and lean mass comprised approximately 20–25% of this reduction. Similarly, another RCT [123] demonstrated a 3.4 kg lean mass decrease in a longer treatment period of 68 weeks using semaglutide 2.4 mg, again reflecting 25% of the 13.3 kg total weight loss, confirming the observation that a longer treatment duration may lead to greater lean mass loss. Although exenatide was less frequently studied, two independent RCTs have provided data regarding anthropometric parameters. In Yin et al. [115] and Bunck et al. [124] studies, lean mass loss ranged from 0.9 to 1.6 kg over treatment periods of 16 to 48 weeks. These studies involved patients with T2D and demonstrated that lean mass preservation was similar to that seen with newer agents, though total weight loss was more modest. We shall point out that although absolute reductions in lean mass tend to increase with the magnitude of total weight loss [125,131], the relative proportion of lean tissue loss remains largely stable—typically representing 20–30% of total body weight reduction across trials. This pattern indicates that GLP-RAs primarily mobilize adipose depots rather than inducing disproportionate skeletal muscle catabolism. Nevertheless, maintaining muscle quality and function remains a clinical priority, particularly in older or metabolically vulnerable populations.

In terms of dual agonists, tirzepatide was evaluated in Heise et al. [116] and Jastreboff et al. [125] studies. In the SURMOUNT-1 trial [125] weight loss approached 22.5% of baseline body weight, with lean mass reduction comprising 26% of the total loss. The average lean mass loss in these studies ranged from 1.9 to 2.8 kg, consistent with the greater potency of tirzepatide in driving overall weight loss.

Importantly, data regarding objective measures of muscle strength, function or performance are scarce [132], limiting interpretation regarding the clinical relevance of body composition changes. Of importance, a recent post hoc analysis of a multicentre open-label RCT (SURPASS-3), where 246 individuals with T2D received either tirzepatide or insulin glargine [127] assessed the use of GLP-1 RA on muscle composition in patients with T2D, overweight and obesity. Tirzepatide treatment was associated with significant reductions in muscle fat infiltration (by 4.4%) and muscle volume, whereas treatment with insulin degludec was associated with a small but significant increase in muscle volume, and no significant changes in muscle fat infiltration. These observations with tirzepatide occurred in the context of significant bodyweight reduction, indicating a qualitative improvement in muscle composition. This finding is of particular interest given the anabolic suppression and intramyocellular lipid accumulation frequently observed in T2D. Consistently, Pandey et al. conducted a pre-specified secondary analysis of a previously published RCT [6] that assessed the treatment with liraglutide 3 mg vs. placebo for 40 weeks on 128 obese female patients without baseline T2D. This study [132] showed that compared to placebo, liraglutide reduced both thigh muscle fat by 7.8% from baseline and adverse muscle composition, defined as high muscle fat and low muscle mass, compared to placebo group.

To this end, an ongoing open-label RCT [133] is assessing the effect of semaglutide in physical function and body composition on older (>65 years) adults with overweight and insulin resistance and similar data on this direction are much awaited. Another prospective study showed that even though treatment with semaglutide in T2D patients resulted in decreased weight, fat mass index and visceral adipose tissue, it preserved muscle strength and muscle quality index after six months compared to baseline [134]. It is of importance to acknowledge that since T2D is a well-recognized risk factor for muscle loss, it is conceivable to assume that by improving glycaemic control and reducing glucotoxicity, GLP-1RAs may indirectly protect skeletal muscle integrity [135]. Moreover, these agents appear to reduce ectopic lipid accumulation within muscle tissue, thereby improving both muscle quantity and quality [136]. GLP-1RAs also promote muscle anabolism through mechanisms involving enhanced vascular perfusion, increased glucose uptake, and activation of the AMP-activated protein kinase (AMPK) pathway, which together stimulate protein synthesis and inhibit proteolytic processes [136,137].

Of importance, in a prospective 6-month study by Peralta-Reich et al. [126], 200 adults with overweight or obesity were treated with either semaglutide or tirzepatide alongside structured resistance training and dietary protein guidance. Over six months, total weight loss averaged 11–13%, yet lean mass loss was limited to approximately 0.63 kg in women and 1.0 kg in men, equating to less than 10% of total weight. Moreover, a recent study in individuals with prediabetes and obesity demonstrated that treatment with tirzepatide does not adversely affect physical function; however, combined resistance and aerobic exercise produced superior improvement in muscle strength compared to tirzepatide on its own [138], underscoring the need for further investigation into integrated therapeutic approaches. These findings underscore the potential of lifestyle co-interventions to mitigate muscle catabolism during pharmacologically induced weight loss, a strategy that may be more effective in younger individuals.

In line with this objective, the EMBRAZE Phase 2 trial investigated the combination of tirzepatide with apitegromab, a highly selective myostatin inhibitor (NCT06445075). While tirzepatide monotherapy was associated with lean mass losses comprising up to 30% of total weight loss, the addition of apitegromab resulted in the preservation of approximately 1.9 kg of lean mass, effectively reducing muscle loss by over 50%. Although still in early development, such combinatorial strategies represent a novel frontier in mitigating the catabolic effects of intense pharmacologic weight reduction. Further developments include the REDEFINE 1 and 2 trials, which evaluated the dual agonist cagrilintide, a long-acting analogue of amylin, with semaglutide (CagriSema) in over 3400 participants [129]. While detailed body composition data are pending, the trials reported unprecedented total weight reductions approaching 20.4%. Given the magnitude of this effect, the forthcoming analysis of lean mass dynamics will be pivotal in assessing the overall metabolic benefit of this therapy. Similarly, phase III data from mazdutide, a GLP-1/glucagon dual agonist, demonstrated significant total weight loss in a dose-dependent manner in Chinese population with overweight or obesity [130]. However, lean mass outcomes remain to be reported.

## 7. Limitations and Future Perspectives

The available evidence undoubtfully suggest that GLP-1 RAs and newer dual or triple incretin agonists deliver profound and sustained weight loss [123,125]. However, their specific effects on lean and fat mass as well as muscle strength and function remain incompletely characterized. Of importance, the research on these effects faces some limitations. A first limitation is the heterogeneity of measurement techniques across studies. Trials employ different modalities, namely DXA, bioelectrical impedance analysis, CT or MRI, often with different analytic approaches and reporting metrics. Some report absolute lean-mass change in kilograms, others use percentages or relative contribution to total weight loss, while hydration adjustments are seldom performed. These methodological differences make cross-trial comparison challenging and contribute to the uncertainty about the magnitude of lean-mass change. Furthermore, the almost exclusive reliance on body-composition surrogates without parallel assessment of functional endpoints. Lean-mass decline does not necessarily equate to a clinically meaningful loss of muscle strength or performance, yet very few trials incorporated measures such as grip strength and gait speed [127,132], a fact largely attributed to the lack of relevant data. However, this is a great filed for ongoing and future research [134]. Another point of concern is that the follow-up in most studies ranges from 24 to 72 weeks, leaving open questions about the extended long-term trajectory of lean mass during chronic therapy or following discontinuation. However, a consistent pattern indicates that longer durations of pharmacotherapy are associated with greater overall weight loss, accompanied by proportionally larger reductions in lean mass. Moreover, the influence of dietary factors, lifestyle parameters and medication other than weight lowering or anti-T2D, has not be adjusted for, and consequently potential biases may be hindered. In addition, trial populations have varied widely, encompassing individuals with and without T2D, different baseline of muscle dysregulations and adiposity levels, and broad age ranges. This heterogeneity raises the possibility that effects on lean mass may differ substantially across subgroups such as older adults, women, or those with pre-existing sarcopenia. Lastly, some studies had a relatively small sample size and therefore the statistical power may not be adequate to distinguish a difference between the experimental and control group.

The afore-mentioned limitations highlight several key research priorities. Evidence indicates that lean-mass losses accompany weight reduction with GLP-1-based therapies, and meta-analyses suggest that lean mass typically accounts for approximately 20–30% of total weight loss, though the proportion varies across trials [123]. It is essential to clarify whether these reductions are merely proportional to overall weight loss or reflect direct drug-specific effects on muscle metabolism, as recent preclinical experimental studies also suggest that GLP-1RAs may positively influence muscle lipid distribution, muscle fibre content [97,98,99], and muscle fibre size [136,137]. Establishing the clinical significance of lean-mass loss is important for both optimal regulation of T2D and for its potential contribution to functional impairment or increased frailty in older or multimorbid patients [119] who may be on incretin-like treatment even for decades. Consistently, this is equally important in MASLD, where long-term alterations in muscle quantity and quality may influence hepatic steatosis progression, insulin sensitivity, and overall metabolic resilience. Comparative studies are needed to define how newer dual and triple agonists affect lean mass relative to traditional GLP-1 monotherapy, given their ability to induce substantially greater weight loss [125,139]. Furthermore, mechanistic investigations should determine whether changes in lean mass are solely a by-product of caloric deficit or whether incretin and glucagon signalling pathways directly modulate muscle proteostasis, mitochondrial function, or intramuscular fat infiltration. Furthermore, emerging data derived from preclinical and clinical studies suggest that the incorporation of resistance training, protein optimization, or adjunctive anabolic therapies can substantially attenuate lean tissue loss [140,141]. Consistently, while the next generation of agents such as CagriSema and mazdutide may achieve superior overall weight loss, their impact on muscle mass must be closely monitored to ensure comprehensive metabolic health and physical resilience. Lastly, the scarcity of comparative data currently prevents a direct comparison between GLP-1 RAs and other approved pharmacotherapies for obesity, including orlistat, phentermine, and topiramate. For clinical practice, these findings emphasize the importance of multimodal treatment plans that integrate pharmacotherapy with exercise and nutritional support, particularly in vulnerable populations.

## 8. Conclusions

Incretin-based pharmacotherapies have transformed the management of obesity and other metabolic comorbidities, delivering clinically meaningful and durable weight loss. However, their effects on lean and fat mass remain insufficiently defined. While GLP-1 RAs and dual/triple agonists inevitably induce some degree of lean mass reduction, this is generally proportional to fat mass loss. Current evidence is limited by methodological heterogeneity, short follow-up, and a lack of functional assessments, leaving uncertainty about the true clinical impact of lean-mass changes.

Future research must address these limitations by implementing standardized and validated methodologies for body-composition assessment, incorporating objective measures of muscle strength and physical performance, and extending longitudinal follow-up to capture long-term trajectories. Rigorous comparative trials of GLP-1RAs, dual GIP/GLP-1 agonists, and emerging triple agonists are warranted to determine whether observed differences in body-composition outcomes are compound-specific or simply proportional to the degree of weight reduction. In parallel, mechanistic studies are needed to elucidate whether these incretin-based therapies exert direct anabolic or catabolic effects on skeletal muscle metabolism independent of caloric restriction. Moreover, future investigations should distinctly evaluate their impact on sarcopenia, SO and myosteatosis to delineate differential effects on muscle quantity, quality, and functional integrity.

Ultimately, designing future trials with muscle health as a central endpoint will be essential. This includes evaluating synergistic strategies that combine pharmacotherapy with resistance exercise, nutritional optimization, or anabolic interventions, particularly in vulnerable populations such as older adults or individuals with SO. Future research should focus on standardizing body composition assessments, evaluating long-term functional outcomes and exploring combinations of metabolic and muscle-targeted therapies. The preservation of lean mass should be considered not merely a secondary endpoint but a primary goal in the pursuit of safe and sustainable weight loss. Only with this comprehensive approach can the full benefits of incretin-based therapies be realized, ensuring that reductions in adiposity do not come at the expense of functional capacity or long-term health.

## Figures and Tables

**Table 1:** Overview of the definition, main characteristics and clinical implications of sarcopenia, sarcopenic obesity (SO) and myosteatosis and their associations with metabolic syndrome and metabolic dysfunction-associated steatotic liver disease (MASLD).

Condition	Definition/Clinical Features	Key Mechanisms	Metabolic and Hepatic Consequences	Associated Biomarkers/Imaging Findings	Clinical Implications
Sarcopenia	Progressive loss of muscle mass, strength, and function; prevalent in T2D, obesity, and older adults	↓ Insulin signalling (Akt/mTOR), mitochondrial dysfunction, ↑ ROS, ↑ TNF-α, IL-6, ↓ myokines (irisin, myostatin imbalance)	↓ Glucose disposal, ↑ insulin resistance, lipid accumulation in liver and muscle	DXA or CT showing ↓ appendicular lean mass; ↓ grip strength or gait speed	↑ Risk of MASLD progression, frailty, falls, and mortality
Sarcopenic Obesity	Coexistence of sarcopenia and excess adiposity; often central/visceral fat accumulation	Chronic low-grade inflammation, ↑ FFAs, ↑ leptin and resistin, ↓ adiponectin, mitochondrial stress	Synergistic impairment in glucose and lipid metabolism, ↑ oxidative stress, endothelial dysfunction	↓ skeletal muscle-to-visceral fat ratio (SVR), ↑ fat-to-lean ratio	↑ Risk of advanced fibrosis, cardiovascular disease, renal dysfunction, and mortality
Myosteatosis	Ectopic fat infiltration in muscle (inter- and intramyocellular)	Lipotoxicity, ceramide accumulation, mitochondrial ROS, impaired β-oxidation, hyperinsulinemia	Worsened insulin resistance, hepatic steatosis, and inflammation; precedes sarcopenia	CT or MRI: ↓ muscle attenuation (Hounsfield units); ultrasound fat fraction >5%	Early marker of MASH and fibrosis; predictive of poor post-surgical and long-term metabolic outcomes

↑: high or upregulation; ↓: low or downregulation. Abbreviations: Akt, Protein kinase B (a key mediator of insulin signalling); CT, Computed tomography; DXA, Dual-energy X-ray absorptiometry; FFAs, Free fatty acids; IL, Interleukin; IR, Insulin resistance; MASH, Metabolic dysfunction-associated steatohepatitis; MASLD, Metabolic dysfunction-associated steatotic liver disease; mTOR, Mechanistic target of rapamycin; MRI, Magnetic resonance imaging; ROS, Reactive oxygen species; SVR, Skeletal muscle-to-visceral fat ratio;; TNF-α, Tumour necrosis factor alpha.

**Table 2:** Pathophysiological mechanisms underlying the effects of GLP-1, GIP, and glucagon on skeletal muscle, lean mass, and body composition.

Hormone/Receptor	Primary Source & Receptor Distribution	Metabolic & Cellular Mechanisms	Effects on Muscle Mass and Lean Tissue	Effects on Adipose Tissue and Body Composition
GLP-1 (Glucagon-like peptide-1)	Secreted by intestinal L-cells; GLP-1R expressed in pancreas, skeletal muscle, heart, adipose tissue, CNS	Activates GLP-1R → ↑ cAMP → PKA and PI3K/Akt signaling Enhances AMPK activity and mitochondrial biogenesis Reduces inflammation and oxidative stress Promotes myogenic differentiation (↑ MyoD, myogenin) Suppresses ActRII/myostatin catabolic signaling	Preserves muscle fiber architecture and contractility in DIO and T2D models Stimulates myogenesis and reduces proteolysis Mild or no net loss of lean mass despite total weight reduction	Preferential reduction in adipose tissue (visceral and subcutaneous) Improves insulin sensitivity and lipid oxidation Decreases ectopic fat (hepatic and intramuscular)
GIP (Glucose-dependent insulinotropic polypeptide)	Secreted by K-cells of duodenum and proximal jejunum; GIPR expressed in pancreatic β-cells, adipocytes, bone, and CNS	Increases insulin and glucagon secretion depending on glycaemic state Enhances lipid storage and adipocyte perfusion Activates PI3K/Akt, cAMP, and MAPK signaling Modulates mitochondrial function and endothelial regeneration	Limited direct anabolic effect on skeletal muscle May indirectly support muscle through improved insulin sensitivity and energy availability	Promotes healthy adipose expansion under physiological conditions May contribute to adipose inflammation under chronic metabolic stress GIPR agonism (as co-agonist with GLP-1) improves fat redistribution and reduces ectopic lipid deposition
Glucagon (GCG)	Secreted by pancreatic α-cells; glucagon receptors (GCGR) expressed in liver, adipose tissue, and skeletal muscle	Activates adenylate cyclase → ↑ cAMP → PKA and CREB Stimulates hepatic gluconeogenesis and lipolysis Increases energy expenditure via brown adipose thermogenesis Enhances fatty acid oxidation and mitochondrial activity in muscle	Promotes mild catabolic effect on muscle in hyperglucagonemia In controlled pharmacologic agonism, may enhance oxidative metabolism without major muscle loss Combined GLP-1/GCG agonism mitigates catabolic risk	Increases lipid oxidation and energy expenditure Reduces hepatic steatosis and visceral adiposity Synergistic with GLP-1 and GIP co-agonists for weight loss with relative lean mass preservation
Dual/Tri-agonists (GLP-1/GIP ± GCG)	Engineered co-agonists acting at multiple receptors	Integrates GLP-1’s insulinotropic and myogenic actions with GIP’s lipid-modulating and GCG’s thermogenic effects Enhances AMPK, PI3K/Akt, and PKA signaling pathways Improves mitochondrial function and substrate utilization	Greater reduction in fat mass with relative lean mass preservation compared to monotherapy Improves muscle metabolic efficiency and reduces ectopic fat infiltration	Synergistic improvement in total and visceral adiposity Increases resting energy expenditure Maintains muscle quality and strength

↑: high or upregulation. Abbreviations: AMPK, AMP-activated protein kinase; Akt, Protein kinase B; ActRII, activin receptor type II; cAMP, cyclic adenosine monophosphate; CREB, cAMP response element-binding protein; DIO, diet-induced obesity; GCGR, glucagon receptor; GIP, glucose-dependent insulinotropic polypeptide; GIPR, GIP receptor; GLP-1, glucagon-like peptide-1; GLP-1R, GLP-1 receptor; MAPK, mitogen-activated protein kinase; MyoD, myogenic differentiation factor D; PKA, protein kinase A; PI3K, phosphoinositide 3-kinase; T2D, type 2 diabetes.

**Table 3:** Randomized clinical trials (RCTs) that evaluated the potential effect of GLP-1 RAs and double/triple incretin agonists on body composition.

Study, Year	Intervention	Duration (Weeks)	Indication	Sample Size	Lean Mass Change (kg)	Assessment Method	Active Comparator	Total Weight Loss (kg)	Fat Mass Change (kg)	Key Findings
Astrup A, 2012 [105]	Liraglutide 1.2–3.0 mg	20	Obesity	371	−1.5	DXA	Placebo	~8.0	~6.5	Lean mass loss ~18% of total; mostly fat loss
Gibbons C, 2021 [106]	Oral Semaglutide 14 mg	12	T2D	15	–	BIA	Placebo	Not reported	Not reported	Short trial; limited lean mass data
Harder H, 2004 [107]	Liraglutide 0.6 mg	8	T2D	21	−0.9	DXA	Placebo	~3.2	~2.3	Modest weight loss; lean mass preserved relatively
Ghanim H, 2020 [108]	Liraglutide 1.8 mg	26	T1D	37	−1.1	BIA	Placebo	~5.0	~3.9	Consistent lean/fat loss proportion
Neeland IJ, 2021 [6]	Liraglutide 3.0 mg	49	Obesity	73	−2.3	DXA	Placebo	~8.5	~6.2	Long duration; lean loss ~27% of total
Ishøy PL, 2017 [109]	Exenatide 2.0 mg	13	Obesity	20	−0.8	BIA	Placebo	~2.7	~1.9	Short-term loss; limited impact on lean mass
Dubé MC, 2018 [110]	Liraglutide 1.8 mg	24	T1D	15	−1.4	DXA	Placebo	~6.0	~4.6	Liraglutide preserved LBM in T1D
Mensberg P, 2017 [111]	Liraglutide 0.6 mg	16	T2D	17	−0.7	BIA	Placebo	~3.5	~2.8	Lean/fat mass loss ratio consistent
Silver HJ, 2023 [112]	Liraglutide 1.8 mg	14	Obesity/Prediabetes	44	−1.2	DXA	Placebo	~4.9	~3.7	Lean loss minimized with support
van Eyk HJ, 2020 [113]	Liraglutide 1.8 mg	26	T2D	22	−1.0	DXA	Placebo	~4.6	~3.6	Liraglutide modestly reduced lean mass
Feng WH, 2019 [114]	Liraglutide 1.8 mg	24	T2D/NAFLD	29	−1.3	DXA	Placebo	~6.0	~4.7	NAFLD patients; body comp improved
Yin TT, 2018 [115]	Exenatide 10 μg	16	T2D	19	−0.9	BIA	Placebo	~4.2	~3.3	Exenatide modest weight & lean mass loss
Heise T, 2023 [116]	Semaglutide 1 mg/Tirzepatide 15 mg	28	T2D	44	−1.9	DXA	Semaglutide	~7.3	~5.4	Tirzepatide had higher loss than semaglutide
Jendle J, 2009 [117]	Liraglutide 0.6–1.8 mg	26	T2D	95	−1.0	DXA	Placebo	~4.5	~3.5	All weight components reduced
Kadouh H, 2020 [118]	Liraglutide 3.0 mg	16	Obesity	19	−1.1	DXA	Placebo	~6.5	~5.4	Lifestyle support attenuated lean loss
Lundgren JR, 2021 [119]	Liraglutide 3.0 mg	48	Obesity	49	−2.0	DXA	Placebo	~7.9	~6.1	Longer duration = greater absolute LBM loss
Grannell A, 2021 [120]	Liraglutide 3.0 mg	16	Obesity	59	−1.8	DXA	Placebo	~7.5	~5.7	Relative lean loss ~24%
Blundell J, 2017 [121]	Semaglutide 1.0 mg	12	Obesity	28	−1.3	DXA	Placebo	~5.6	~4.3	Proportional lean/fat mass change
McCrimmon RJ, 2020 [122]	Semaglutide 1.0 mg	52	T2D	88	−1.7	BIA	Placebo	~10.0	~8.3	Substantial weight/fat loss; lean preserved
Wilding JPH, 2021 [123]	Semaglutide 2.4 mg	68	Obesity	95	−3.4	DXA	Placebo	~13.3	~9.9	Lean mass loss ~25% of total
Bunck MC, 2010 [124]	Exenatide 20 μg	48	T2D	29	−1.6	DXA	Insulin glargine	~4.0	~2.4	Insulin glargine vs. Exenatide showed similar LBM
Jastreboff AM, 2022 [125]	Tirzepatide 5–15 mg	72	Obesity	255	−2.8	DXA	Placebo	~15.0	~12.2	High % lean mass loss (~26%)
Peralta-Reich et al., 2025 [126]	Semaglutide or Tirzepatide + lifestyle	24	Obesity	200	~−0.63 (F); ~−1.0 (M)	BIA	Self	~11.0	~10.0	Exercise/protein support preserved lean mass
Glasgow Univ., 2025 [127]	Tirzepatide	52	T2D	246	Preserved	MRI/CT	Insulin glargine	~13.0	~13.0	Intramuscular fat ↓; mass preserved
Scholar Rock, 2025 (NCT06445075) [128]	Tirzepatide ± Apitegromab	24	Obesity	—	−30% (tirz); +1.9 kg w/Apit	—	Tirzepatide	~15.0	~15.0	Apitegromab preserved 1.9 kg LBM
REDEFINE 1/2, 2025 [129]	CagriSema	68	Obesity	>3400	Pending	—	Placebo	~20.4	Pending	CagriSema produced highest total WL
Mazdutide Phase III, 2025 [130]	Mazdutide	—	Obesity	—	Not reported	—	—	~15.0	Not reported	Awaiting lean mass outcomes

↓: low or downregulation. Abbreviations: BIA, bioelectrical impedance analysis; CT, computer tomography; DXA, Dual-energy X-ray Absorptiometry; LBM, lean body mass; MRI, magnetic resonance imaging; T1D, type 1 diabetes; T2D, type 2 diabetes.

## Data Availability

No new data were created or analyzed in this study. Data sharing is not applicable to this article.
